# Direct observation shows superposition and large scale flexibility within cytoplasmic dynein motors moving along microtubules

**DOI:** 10.1038/ncomms9179

**Published:** 2015-09-14

**Authors:** Hiroshi Imai, Tomohiro Shima, Kazuo Sutoh, Matthew L. Walker, Peter J. Knight, Takahide Kon, Stan A. Burgess

**Affiliations:** 1 School of Molecular and Cellular Biology and Astbury Centre for Structural Molecular Biology, Faculty of Biological Sciences, University of Leeds, Leeds LS2 9JT, UK; 2 Quantitative Biology Center, Riken, 6-2-3 Furuedai, Suita, Osaka 565-0874, Japan; 3 Faculty of Science and Engineering, Waseda University, Takada 1-17-22, Toshima-ku, Tokyo 171-0033, Japan; 4 MLW Consulting, 11 Race Hill, Launceston PL15 9BB, Cornwall, UK; 5 Department of Biological Sciences, Graduate School of Science, Osaka University, 1-1 Machikaneyama-cho, Toyonaka, 560-0043 Osaka, Japan; 6 Japan Science and Technology Agency, Precursory Research for Embryonic Science and Technology, Kawaguchi, 332-0012 Saitama, Japan

## Abstract

Cytoplasmic dynein is a dimeric AAA^+^ motor protein that performs critical roles in eukaryotic cells by moving along microtubules using ATP. Here using cryo-electron microscopy we directly observe the structure of *Dictyostelium discoideum* dynein dimers on microtubules at near-physiological ATP concentrations. They display remarkable flexibility at a hinge close to the microtubule binding domain (the stalkhead) producing a wide range of head positions. About half the molecules have the two heads separated from one another, with both leading and trailing motors attached to the microtubule. The other half have the two heads and stalks closely superposed in a front-to-back arrangement of the AAA^+^ rings, suggesting specific contact between the heads. All stalks point towards the microtubule minus end. Mean stalk angles depend on the separation between their stalkheads, which allows estimation of inter-head tension. These findings provide a structural framework for understanding dynein’s directionality and unusual stepping behaviour.

## Abstract (web summary)

Cytoplasmic dynein is a dimeric protein that steps processively along microtubules. Here Imai *et al*. present cryo-electron microscopy images of stepping *D. discoideum* dynein, revealing diverse microtubule-bound configurations including a hinge-dependent, motors side-by-side arrangement.

## Graphical abstract

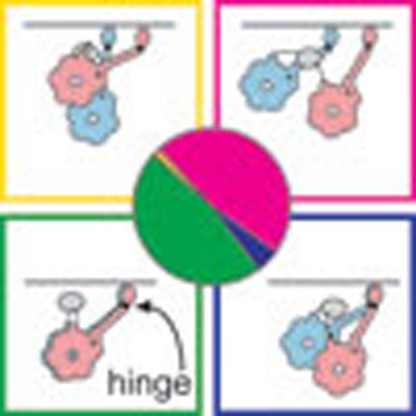

Dyneins are a group of motor proteins that move along microtubules (MTs) to cause the beating of the axoneme in cilia and flagella and to perform vital and diverse transport and tethering roles in the cytoplasm of eukaryotic cells, for instance transporting mRNA, growth factors and β-amyloid precursor protein^1^,^2^. Dynein also transports the nucleus in neurons, which is essential to human development and maintenance of healthy neuronal activities^3^,^4^. Growing numbers of neurodegenerative diseases and developmental problems are now known to result from mutations in dynein or dynein-binding proteins^5^,^6^, and dynein-mediated processes are implicated in cancer^7^. So, in addition to its intrinsic interest, understanding dynein mechanism is critical for future treatment of disease.

Dyneins have an unusual structure in which each ring-like ATPase head attaches to the MT via a slender, coiled-coil stalk at the tip of which is a small, globular, MT-binding subdomain that we term the stalkhead. (The stalkhead has also been called the MT binding domain (MTBD) but the meaning of MTBD is ambiguous for axonemal dyneins, since the stalkhead binds to one MT doublet and the tail binds to an adjacent doublet; by contrast, ‘stalkhead’ is intuitively understood and more concise.) The atomic structure of the motor domain is known^8^,^9^,^10^. The heart of the motor domain is a AAA^+^ superfamily mechano-enzyme^11^, in which six AAA^+^ motifs form a ring that hydrolyses ATP^1^. Associated with the AAA^+^ ring (hereafter simply ‘ring’) is a C-terminal sequence that is implicated in determining the stall force and run length of the motor^12^,^13^, and that is unusually short in the much-studied yeast dynein^13^,^14^. Cytoplasmic dynein includes two identical heavy chains, each forming tail and motor domains (Fig. 1a)^1^. The complex N-terminal tail incorporates additional polypeptides, binds cargoes and dimerizes the motors^15^,^16^,^17^. Between the tail and ring is the linker^18^ that provides the power stroke of the motor^19^ by switching from bent (primed) to straight (unprimed)^8^,^9^,^10^,^20^. The ring, C-terminal sequence and linker together constitute the head, from which the stalk extends (Fig. 1a)^21^.

Individual cytoplasmic dynein dimers can make runs of many steps along a MT, that is, they are processive. For many dyneins, the processive molecule includes a complex between the tail and other proteins or protein complexes such as BicD and dynactin^22^,^23^. For yeast and *Dictyostelium* dyneins, replacement of the tail with glutathione S-transferase (GST; Fig. 1b) yields a simpler dimer that still processively steps along MT^13^,^24^,^25^. The structural mechanism of cytoplasmic dynein’s processive stepping along MTs is unclear. Tracking fluorescently-tagged dynein suggested uncoordinated stepping by the two heads^26^,^27^, but they must also communicate since the properties of dimers on the MT are different from those of monomers^13^,^28^. Dynein’s unusual architecture suggests that its stepping mechanism may be fundamentally different from the other transport motors, kinesin and myosin.

The structural basis of processive stepping by cytoplasmic dynein has not previously been investigated. Understanding of myosin-5’s movement along actin filaments was greatly aided by visualizing it on its actin track by using negative stain electron microscopy at rate-limiting (micromolar) ATP concentrations^29^. Such a study on dynein would be problematic because MT diameter is much greater than actin filament diameter, which could lead to structural collapse of both MT and dynein during drying of the stained specimen. Therefore, to understand dynein stepping, we have used the technically more demanding unstained cryo-electron microscopy (cryo-EM) technique to discover the structures of stepping dynein. Unlike many recent applications of cryo-EM, our aim here is not to produce a high resolution structure of the stepping molecule, but rather to trap a dynamic motor in action, so that we can see the range of structures that dynein adopts. We have succeeded in imaging dynein-MT complexes at near-physiological (millimolar) ATP and revealed a great diversity of structures.

## Results

### Cryo-EM of cytoplasmic dynein dimers moving on MTs in physiological ATP

**Figure 1:**
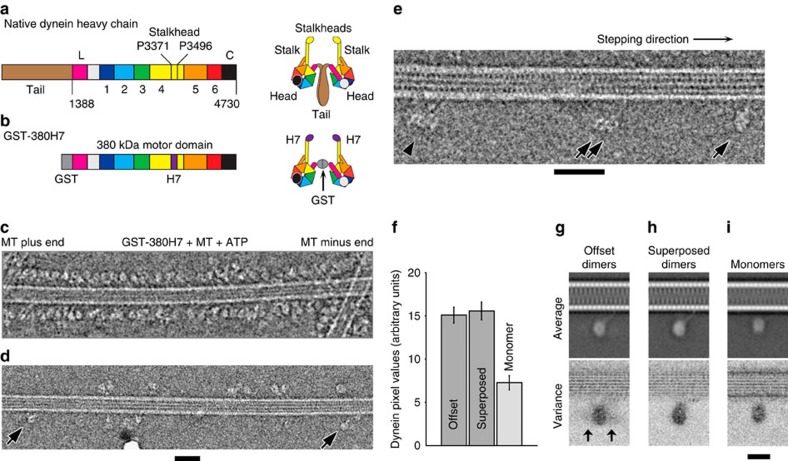
Cryo-EM of dimeric dynein motors bound to MTs in 2 mM Mg-ATP. (**a**) Domains in the amino acid sequence of *D. discoideum* dynein heavy chain and (right) cartoon depicting the domain architecture of the dimer. The N-terminal tail (residues 1-1387, brown) dimerizes the motor *in vivo*. The motor includes six AAA^+^ modules: AAA1 (blue)-AAA6 (red). Stalk and stalkhead emerges from AAA4, the strut from AAA5 and sequence numbers indicate the invariant prolines at the stalk-stalkhead junction. Linker (magenta and pale grey) and C-terminal domain (black) lie on opposite faces of the AAA^+^ ring. Proposed moving part of the linker (L, magenta) is indicated. (**b**) Recombinant chimeric dynein dimerized by GST and with stalkhead from human axonemal dynein heavy chain 7; (right) cartoon depicting this dimer. (**c**–**e**) Cryo-EM of GST-380H7 mixed with MT and ∼2 mM Mg-ATP. Contrast is inverted (protein is pale) and dynein’s stepping direction (to the MT minus end) is towards the right in all Figures. (**c**) Dynein particles are crowded along the sides of the MT; such regions were not further analysed. (**d**) In sparse regions of less densely decorated MTs can be seen ‘superposed’ dimers (arrows) in which only a single ring is visible. (**e**) Enlarged view showing ‘superposed’ dimer (single arrow), an ‘offset’ dimer in which both rings are visible (double arrow) and a group of dimers comprising more than two rings (arrowhead). (**f**) Pixel values calculated from the head domains relative to adjacent MT (see Supplementary Fig. 4) of offset and superposed dimers and of monomers (mean±s.e.m.). (**g**,**h**) Image analysis of dimers stringently isolated on the MT surface (see text). Average (upper panel) and variance (lower panel) images of MT-aligned offset dimers (**g**) and superposed dimers (**h**) with MT at the top. Higher variance is darker grey. In offset dimer average (**g**) only one ring is apparent but the second ring’s variable position is revealed in the variance image (arrows). (**i**) Monomeric dynein bound in the absence of nucleotide. Average appears closer to MT than dimers because some particles were attached to MT protofilaments lying closer to the MT axis in this view. Scale bars, (**c**–**e**) 40 nm; (**g**-**i**) 20 nm.

In the presence of millimolar MgATP, we found most artificially-dimerized *Dictyostelium discoideum* cytoplasmic dynein motors (GST-380) are detached from MTs. Replacement of the stalkhead with that of human axonemal dynein 7 (DNAH7) to form GST-380H7 produces a dimer that has a similar structure in the absence of MTs as GST-380 (Supplementary Fig. 1), but stronger binding to MTs in TIRF-M stepping assays (Supplementary Movie 1). The longer duration of runs together with the higher attachment rate of GST-380H7 compared with that of GST-380 also indicate that GST-380H7 has a much higher affinity to MTs than GST-380 (Supplementary Table 1), and this chimeric dynein moves robustly along MTs (Supplementary Fig. 2a; Supplementary Movie 2). Cryo-EM in the presence of ∼2 mM Mg-ATP shows almost all GST-380H7 dimers are bound to the MTs (Fig. 1c–e), including at their flared ends (Supplementary Fig. 2). Dynein forms a fringe along either side of the MT, not all around it (Fig. 1c), indicating that these rapidly stepping molecules use the few seconds taken to prepare the specimen to favour the MT protofilaments nearest the centre of the thin (typically 50 nm) liquid film. This simplifies the analysis of the configurations of the stepping dynein molecules.

### Characterization of MT-bound dimer configurations

Raw images have sufficient contrast to show the head and stalk domains (Fig. 1d, arrows), but greater structural detail is revealed using single-particle image processing^30^. We first determined MT polarity^31^ and hence the direction of dynein stepping (see Supporting Methods). From 1,082 such MTs we identified 10,080 dynein-MT complexes. To avoid ambiguity of assignment of heads into dimers we then selected a subset of isolated molecules in which two heads <40-nm apart lacked neighbours within ±40 nm along the MT. Forty nanometres exceeds the maximum head–head separation observed in MT-free GST-dynein dimers (Supplementary Fig. 1c). This stringent criterion yielded 374 molecules showing two variably spaced heads, referred to as offset dimers. A second group of 322 molecules showed only a single head >40 nm from its nearest neighbour (single arrows, Fig. 1d,e). For such heads, summed, normalized pixel values of individual molecules are almost twice those of monomeric dynein (that is, lacking the N-terminal GST) bound to MTs, and are within error the same as those of the combined heads of offset dimers (Fig. 1f; method in Supplementary Fig. 4). This shows that such heads are dimers with superposed heads, not monomers. Thus GST-380H7 dimers stepping along MT adopt a variety of configurations, including an abundant one in which the two heads are very closely superposed.

Global averages of MT-aligned offset and superposed dimers show indications of the stalk binding to the MT, oriented at a moderate angle to the MT surface and pointing towards the minus end of the MT (Fig. 1g,h). However, the second head of offset dimers is smeared out and structural details are obscured, partly because dynein’s head position is a variable distance from the MT surface. To see detail and quantify this flexibility, we further analysed superposed and offset configurations.

### Structure of the superposed dimer

**Figure 2:**
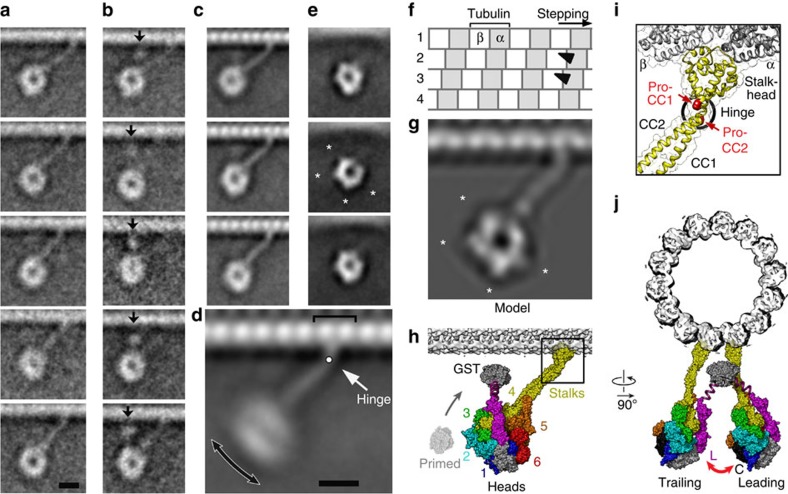
Structure and flexibility of superposed dimers. (**a**,**b**) Averaged images of superposed dimers from sparse regions. (**a**) Classified to reveal the rings and stalks connecting the motor to the track. Stalk flexibility alters the distance between the rings and MT surface. (**b**) Classified to reveal the GST (arrows) variably positioned between the ring and the MT surface. Number of images in each class varies between 10 and 23. (**c**–**e**) Averaged images of dimers from crowded regions. (**c**) Well-populated classes show the stalk and stalkheads clearly despite stalk flexibility. (**d**) Combined images including those from (**c**) shows flexibility occurs mainly in the plane of the image (double-headed arrow). Hinge between stalk and stalkhead (white arrow) and binding site at the interface of tubulin subunits (bracket) are indicated. (**e**) Class averages of aligned head domains showing the ring substructure. Prominent peripheral features (asterisks in middle panel) are compatible with features (asterisks) seen in computed projections (**g**) of a model (**h**) of the ADP-dynein superposed dimer bound to the MT (chimera of dynein motor from PDB 3VKG and stalkhead from PDB 3J1T; see Methods and Supplementary Figs 5–7). (**f**) Diagram depicting four MT protofilaments, staggered axially by 0.9 nm, each comprising dimers of α- (grey) and β- (white) tubulin subunits at 8.3-nm intervals. Stalkhead binding sites (black triangles) deduced for the superposed dynein configuration on adjacent protofilaments are illustrated. (**h**) Surface rendered model of the superposed dimer. Motor structure coloured as in Fig. 1a. AAA^+^ domains (1–6) are indicated. The GST dimer is depicted connecting to the linkers by magenta springs. (**i**) Enlargement of boxed (stalkhead) region of (**h**) showing one stalk depicted as backbone ribbons and showing location of invariant prolines P3371 and P3496 (red spacefill) and the position of the flexible hinge (black ring). (**j**) Model in (**h**) viewed looking towards the MT minus end; the front-to-back arrangement of the two motors shows how the linker (L) and C-terminal domain (C) of the two motors face one another (red arrow). Scale bars in (**a**) for (**a**–**c**,**e**), and in (**d**) for (**d**,**f**–**h** and **j**) are 8 nm.

Further alignment of superposed dimers using only features of dynein, shows that they display the structure of a circular ring with pronounced central channel (Fig. 2a). Extensive classifications designed to reveal slight displacements between the two rings did not detect any. Thus, the two rings are accurately superposed and both lie in approximately parallel planes that are also parallel to the MT long axis such that they are seen face-on in this side decoration of MT. The perpendicular distance from the MT surface to the ring centre is 14.4 nm±3.0 nm (mean±s.d., *n*=322).

The GST dimer was located, by classification around the superposed rings, variably positioned between the rings and the MT surface, close to the stalk on the MT plus end side (arrows, Fig. 2b). It is likely that attachment of bulky probes to the GST for stepping studies^25^,^26^,^32^,^33^ sterically hinders this conformation. Because GST marks the end of dynein’s linker, this location means that the linkers must be in front of the rings (that is, nearer the observer) in this view (Fig. 2h)^8^,^9^,^20^. This location also means that both linkers are close to the unprimed conformation (Fig. 2h), which further indicates that both stalkheads of the superposed dimer are bound to the MT, as this linker position is associated with tight binding to MTs^34^.

The MT-bound stalk is clearly visible (Fig. 2). Classification around the superposed rings did not detect a second stalk, indicating that the two stalks are superposed. Binding sites on adjacent MT protofilaments can produce such stalk superposition because protofilaments are staggered axially by only 0.9 nm in a left-handed helix^35^ compared with the ∼2-nm stalk diameter (Fig. 2f). The stalkhead nearer the observer is thus 0.9 nm ahead of the other stalkhead.

Greater detail of the ring, stalk, stalkhead and adjacent MT lattice of the superposed dimer is revealed by selecting ∼1,500 more examples from the full data set (Fig. 2c,e) to improve signal-to-noise in image averages. The superposed stalkheads are attached to the MT at the subunit interface within the tubulin dimer (Fig. 2d) and join the stalk at an abrupt kink (Fig. 2d).

Superposed dimers show striking variation in ring distance from the MT, which arises principally from flexion at the stalk-stalkhead junction (Fig. 2a,c,d; Supplementary Movie 3). Since the rings remain in plane at all angles, this junction acts as a hinge rather than a universal joint, with its rotation axis tangential and orthogonal to the axis of the cylindrical MT. Given both GST and hinge flexibility, it is remarkable that the rings are so often so closely superposed. This suggests that superposition might be maintained by direct head–head interactions.

### Atomic model of the superposed dimer on MT

To gain insights into possible interactions between the two superposed motors and the structural basis of flexibility at the stalk-stalkhead hinge, we used recent structural data^8^,^36^ to build atomic models of the MT-bound superposed dimer to compare with the EM data (Supplementary Figs 5–7). The back-to-back head arrangement found in a *D. discoideum* crystal structure of monomers^8^ corresponds poorly with our data (Supplementary Fig. 7a–c), and the stalkheads face in opposite directions, incompatible with simultaneous binding to the polar MT. Combinations of the two different head-stalk crystal structures provide four distinct front-to-back dimeric models (Supplementary Fig. 6d–g). Such models are compatible with EM data in stalk angle and linker location, but the rings are foreshortened because they are tilted out of the plane of the EM view (Supplementary Fig. 7d–g). Rotating the rings into this plane (Supplementary Fig. 6h) produces a model (Fig. 2h; Supplementary Movie 4) that closely resembles the head structures seen in our data (Fig. 2g cf. Fig. 2c,e). In this configuration, the linker of the motor further from the observer is juxtaposed to the C-terminal domain of the nearer motor (Fig. 2j), suggesting these are the components that might interact to maintain superposition of the rings. It is notable that unlike most dyneins (including human and *D. discoideum* dyneins), the much-studied yeast dynein lacks most of the C-terminal domain^13^,^14^, so such contacts, if present, would be different.

The atomic model of dynein on MT shows that the stalk-stalkhead hinge coincides with the location of the invariant proline residues^14^ in the stalk (Fig. 2i). Here the two stalk helices are superposed in this view, which allows hingeing in the plane observed without stretching the helices. Flexion at the same position and in the same plane was also found in molecular dynamics simulations of the stalkhead plus stalk in the absence of MT^37^, which suggests that the behaviour of this hinge is qualitatively independent of strong attachment of the stalkhead to MT.

### Structure and diversity of offset dimers

**Figure 3:**
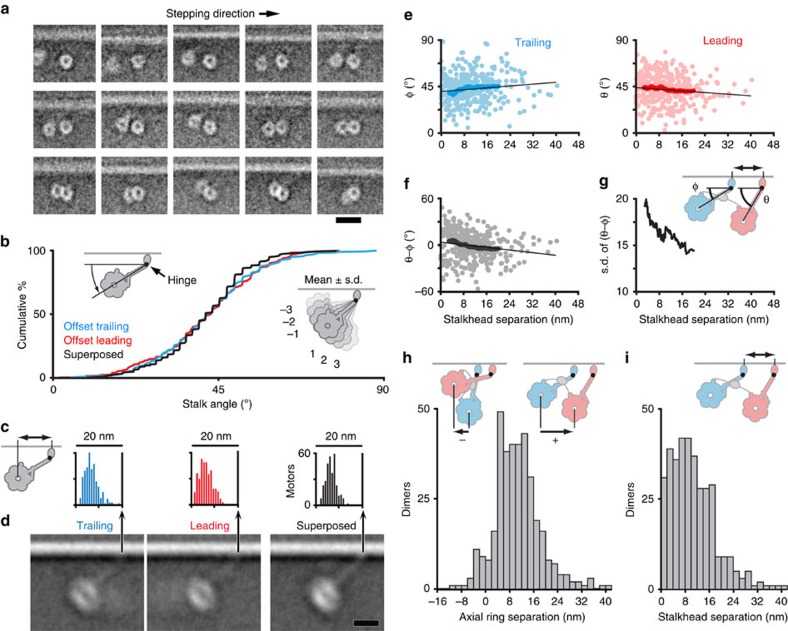
Flexibility and intramolecular tension in offset dimers. (**a**) Example class averages of offset dimers classified to show the relative position of the two rings (see Methods) and arranged in order of decreasing ring–ring separation. The leading ring is shown at a constant position. (**b**) Deduced stalk angle (left cartoon) shown as cumulative percentage plot for trailing (cyan) and leading (red) motors, with superposed dimers (black) for comparison. Right cartoon illustrates the mean angle and the wide spread of head positions due to stalk hingeing, with numbers referring to the angle of each subsidiary motor cartoon away from the mean, expressed as a multiple of s.d. (**c**) Histograms of distance between ring centre and stalkhead position (cartoon). Trailing motor (mean 13.5±3.1 nm s.d.), leading motor (13.6±2.9 nm) and superposed dimer (13.8±2.4 nm). (**d**) Image averages of motors aligned according to stalkhead position, excluding motors with extreme stalk angles for clarity. Images shown at the same scale as histograms in (**c**). (**e**) Scatter plots of stalk angle of trailing and leading motors (see cartoon in (**g**); note GST (grey) connecting the motors) plotted as a function of the separation between stalkheads in each dimer. Also shown, running averages (darker points), using a window size of 100 data points, and linear regression lines (black, trailing: *y*=40.0+0.222x; leading: *y*=44.1−0.200x; *n*=359; slopes significantly different from zero at *P*<0.05, one-tailed *t*-test). (**f**) As (**e**), but depicting difference between leading and trailing stalk angle of dimers. (**g**) s.d. of the running average values shown in (**f**). (**h**) Histogram of axial ring separation measured from trailing to leading ring (cartoons). Most dimers have positive ring separation, others have negative separation, illustrated by the inset cartoons. (**i**) Histogram of stalkhead separation (cartoon). Scale bar, 20 nm (**a**), 10 nm (**d**).

Both rings of offset dimers are revealed by careful alignment and classification (Fig. 3a). They appear circular, with the central channel clearly visible, regardless of ring–ring separation distance. Thus the plane of each ring lies approximately parallel to the axis of the MT, like the rings in superposed dimers. In many dimers the rings partially overlap (Fig. 3a, bottom row) implying that such rings must be offset azimuthally (that is, around the MT axis) to avoid steric clash. GST is not seen, indicating that its position is highly variable within the offset dimer. Variable position of the aligned rings with respect to the MT smears out the MT (cf Fig. 1g) and consequently also the stalks, which are rarely visible in these averages. Nevertheless, the ring-MT distances of both leading and trailing rings are very similar to those of superposed dimers, indicating that both motors are bound to MT (Supplementary Fig. 8).

To visualize the stalks in offset dimers, we surmised that, as in superposed dimers, observed variation in ring-MT distance arises from flexion of the motor as a rigid body at the stalk-stalkhead hinge. The constant distance from ring centre to hinge allows calculation of stalk angle for each motor by trigonometry and thus prediction of stalkhead location on the MT in the minus end direction from the ring (Supplementary Fig. 8a). Alignment of the stalkheads based on this prediction, indeed reveals variably angled stalks with stalkheads attaching both leading and trailing motors to the MT (Fig. 3c,d; Supplementary Movie 5). This confirms that both motors of offset dimers are bound to the MT, and that both stalks point to the minus end. The calculated stalk angles are remarkably similar for leading and trailing motors (41.9°±13.7° and 42.7°±13.7°, respectively, (mean±s.d.; Fig. 3b) as well as for superposed dimers from sparse regions analysed in this way (41.5°±11.5°; *n*=318). The stalk-stalkhead hinge causes the distance the head lies behind the stalkhead to vary over a wide range (∼20 nm; Fig. 3c).

Relative independence of the two motors linked through the GST tether is implied by the broad scatter of stalk angles of leading and trailing motors (Fig. 3e; Supplementary Fig. 8c). Consequently, and because of the wide range of stalk angles, the two stalks of the dimer sometimes cross, in which case the leading stalkhead attaches to the (low angle) trailing ring (Fig. 3h). We define such a motor as a leading motor as it is the stalkhead that is anchored to the MT. The broadly distributed axial separation of the rings thus includes negative values (Fig. 3h).

The axial separation between the stalkheads of offset dimers has a broad distribution (Fig. 3i). The discrete peaks expected from the spacing of MT-binding sites (Fig. 2f) are absent, possibly due to alignment and measurement errors; flexibility elsewhere in the motor that compromises the assumptions of the trigonometric method; and dimers attached to two protofilaments that form a seam in the 14_3 MT^35^ and are thus further offset by ∼4 nm. The distribution includes very small separations indicating that some dimers have superposed stalkheads but offset rings. Most stalkheads are separated by ∼8 nm, fewer by ∼16 nm and few by ∼24 nm.

### Mechanical properties of dynein

The flexibility shown by our images of dynein allows deductions to be made which advance understanding of the mechanical properties of the molecule that underlie its function. The stalk-stalkhead hinge is a major site of compliance within the MT-bound dimer (Supplementary Movies 3 and 5; see also ref. 37). Applying the equipartition theorem to the measured variance in angle^38^ yields estimates of the torsional stiffness for this spring-like hinge in leading, trailing and superposed moieties of 72, 71 and 101 pN nm rad^−2^, respectively. Given that the mean stalk angle is ∼42°, this yields apparent cantilever stiffness of these attached motors (that is, their resistance to axial forces applied at the base of the stalk) of 1.07, 1.03 and 1.53 pN nm^−1^, respectively (see Methods). The higher stiffness of the superposed dimer may be explained by the two springs being connected in parallel through interactions between the superposed heads, rather than being almost independent.

The data provide evidence of mechanical communication between the two offset motors. If stalkheads are attached far apart on the MT, the GST tether should pull the heads together, producing a shallower leading stalk angle and a steeper trailing stalk angle. Such trends are indeed apparent despite the broad scatter: both regression slope and running average of stalk angles show the predicted dependence on stalkhead separation (Fig. 3e), it appears linear and it is similar in magnitude for leading and trailing stalks (−0.20° nm^−1^ and +0.22° nm^−1^, respectively). Likewise, the pairwise difference between leading and trailing stalk angle of a dimer has the predicted negative dependence on stalkhead separation (−0.42° nm^−1^; Fig. 3f). The s.d. of this measurement also markedly decreases with increasing stalkhead separation (Fig. 3g), indicating that the positions of the two heads become more correlated. The regression equations (Fig. 3e) can be used to estimate, for any given stalkhead separation, the mean axial ring centre separation. Thus, stalkhead separations of 0.9, 8.3, 16.6 and 24.9 nm (see Fig. 2f) yield mean ring centre separations of −0.1, 6.6, 14.2 and 21.7 nm, respectively.

The time-averaged tension developed within the offset dimer can be estimated by combining the torsional stiffness of the stalk-stalkhead hinge derived above with the dependence of stalk angle on stalkhead separation. This yields estimates per nm of stalkhead separation of 0.031 and 0.033 pN using leading and trailing motor data, respectively, which averages 0.032 pN. Thus, for dimers with stalkheads separated by 8.3, 16.6 or 24.9 nm, the time-averaged tension would be 0.26, 0.53 or 0.79 pN, respectively. All components of the dynein dimer will experience this tension in series, and thus the stiffness of the elastic linkage between the two rings in this GST dimer can be estimated as 0.035 pN nm^−1^ by dividing the increment of tension by the increment of ring–ring separation (see above). The increase of inter-head tension with step length will bias towards shorter steps.

## Discussion

We succeeded in seeing dimeric dynein attached to MTs in ATP by using *Dictyostelium* cytoplasmic dynein motors dimerized by GST at the end of the linker. They also have the stalkhead replaced by one derived from a human dynein, which increases the fraction of dimers found attached to the MT in ATP by cryo-EM. Several lines of evidence indicate that this construct shares many properties with intact dynein. First, for our construct attached to MT in ATP, the stalk angle and the position of GST, close to the MT surface, are consistent with the stalk angle and the position of the linker-tail junction of intact dynein bound in rigor on MT^39^. Second, in the absence of MT the range of head–head separations allowed by GST dimerization (Supplementary Fig. 1c) is comparable to intact dynein^22^,^23^,^39^,^40^. Third, our constructs form compact phi particles (Supplementary Fig. 1b,e) with a motor arrangement similar to intact dynein^22^,^40^,^41^. Finally yeast GST-dynein movement on MTs is comparable to intact yeast dynein^25^. Thus, there are strong grounds for supposing that our findings are informative for understanding the behaviour of intact cytoplasmic dynein.

Our cryo-EM of stepping dynein reveals a great diversity of structures. Not only is there the variety in the separation of stalkheads along the MT anticipated from step-measuring studies, but also a flexibility at the stalk-stalkhead junction that produces a continuum of structures that is especially marked for offset dimers. We have been able to exploit this flexibility to deduce estimates of mechanical properties of dynein attached to its track. When stalkheads are attached far apart along the MT, the internal stress within the dynein dimer is expressed partly in a change in the angles of both stalks, allowing the heads to be less far apart, but principally by an extension of the linkage between the two heads. We deduce a value of 0.035 pN nm^−1^ for the elastic spring constant of this head–head linkage. Modelling the behaviour of a dimerized yeast dynein^33^ has suggested a similar value (0.083 pN nm^−1^). The molecular basis of this elastic linkage is unclear. If it were an unstructured polypeptide behaving as a worm-like chain, the deduced low stiffness over the wide observed range of head separations would require a polypeptide of several hundred amino acids, which is not present in our dimeric dynein. An alternative which invites future investigation is flexion of the two linker subdomains away from the unprimed conformations that they adopt in the superposed dimer.

**Figure 4:**
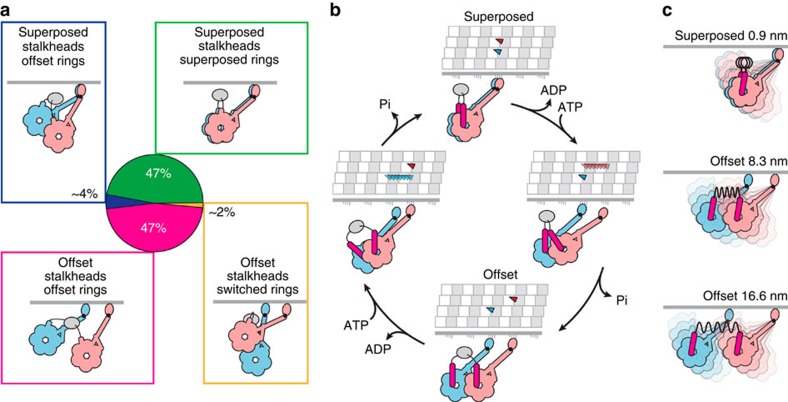
Summary of dimer configurations in ATP and tentative stepping model. (**a**) Dimer configurations identified in this study. GST dimer is shown as grey ellipse. (**b**) An example of a two-step advance along the MT, tentatively correlated with consumption of two molecules of ATP and showing the stalkhead binding sequence both as viewed from the rings and in the view seen in our data. The mobile part of the linker is illustrated (magenta) switching to the primed conformation during stalkhead detachment, as well as a possible trajectory of each detached stalkhead along the MT protofilament. In this example, 8 nm stepping occurs along two adjacent protofilaments and superposed and offset dimers alternate during stepping, illustrated here as an inchworm progression. Examples of alternative stepping patterns are illustrated in Supplementary Fig. 9. (**c**) In superposed dimers, the stalkhead-stalk hinge allows flexing. In offset dimers, the compliant GST-linker bridge between rings (black coil spring) additionally allows quasi-independent flexing of the heads and pulls the heads towards one another with increasing stalkhead separation.

A natural interpretation of our finding that superposed and offset heads are equally abundant is that these structures alternate during stepping and that each has a similar lifetime. An example of how such a stepping cycle could coordinate with ATPase activity and how the two stalkheads would move along the MT is shown in Fig. 4b. Strict alternation is not, however, implied by our data and other examples that demonstrate more freedom, including stepping along single protofilaments and movements between protofilaments, are shown in Supplementary Fig. 9. If the lifetimes of the two structures are unequal, the one with shorter lifetime should occur more often during a stepping sequence to yield the observed similar abundance.

An important parameter for a processive, cargo-carrying motor protein is its duty ratio, that is, the fraction of the motor cycle during which it is strongly attached to its track. The duty ratio of the dimeric dynein that we have imaged is ∼0.9 if determined assuming independent heads^42^ and ∼600-nm mean run length on MT (Supplementary Table 1; see Methods). This is a higher value than our previous estimate (0.6) for dimeric *D. discoideum* dynein^28^, and much higher than that of monomer (0.2) ref. 28. By EM we have not detected dimers with one detached motor, suggesting that each motor is almost always attached, that is, has a duty ratio near 1.

What structural mechanisms could enhance processivity of the dimer over that of monomer to reduce the probability of both heads detaching simultaneously? In the superposed dimer the two motors appear identical, so it is unclear why either motor would behave differently from a monomer, unless this is mediated by contacts between the two heads. In offset dimers, we find that intramolecular force biases the orientation of the two stalks in opposite directions (which has been shown to alter the force required to detach them from the MT^24^,^33^), and this force may have further impacts on head structure, such as linker position.

When dynein is pulling against a load, formation of the superposed dimer may be favoured because the advancing head may more often attach only 0.9 nm ahead of its partner, and the compact structure may then provide additional stability, helping dynein function as an anchor. This reasoning thus predicts that the superposed configuration is dominant in, for example, the mitotic spindle (see ref. 1). The ratio of the frequency of superposed and offset rings among molecules with superposed stalkheads (318/∼31; see Fig. 3i, first bar) yields an estimate of ∼10 for the equilibrium constant for the head–head association when the two motors are attached to the MT under no load, and resistive load would be expected to increase this value.

Our results provide direct evidence for the mechanism of dynein stepping in ATP, since we have observed dynein directly during ATP-fuelled movement along MT. This establishes that (1) the angle between stalk and MT is ∼42° for both leading and trailing motors; (2) the ring of the attached motor maintains a constant orientation in which its plane is parallel to the axis of the MT; (3) the linkers point towards the MT track, rather than away from it (Fig. 4). In our atomic model derived from earlier EM and crystal structure data (Fig. 2h) the stalk angle is similar (∼45°), but steeper angles of 50°-70° are seen for monomeric dynein densely labelling MTs^36^ and for axonemal dyneins^43^,^44^,^45^. With this motor configuration on the MT, and as is shown in Fig. 4b, when ATP binding to the ring causes stalkhead detachment from^46^ (or weak binding to^40^,^47^) the MT, the subsequent linker swing to the primed position is in a direction which biases the stalkhead towards the minus end of the MT, that is, in the known direction of dynein movement. This is because, in order for the stalkhead to rebind stereospecifically to the MT, the stalk must be pointing towards the MT minus end. Rebinding to the MT, followed by linker swing back to the unprimed position (that is, the power stroke), will then drag dynein’s cargo towards the MT minus end.

Perhaps the most striking feature of stepping dynein is the great flexibility between the ATPase domain and the track binding domain (Fig. 4c), which is in marked contrast to myosin and kinesin motors. Because the hinge is close to the MT surface, at the stalk-stalkhead junction, the dynein head swings over a wide range (∼20 nm) compared with the ∼8 nm spacing of binding sites on the MT. This suggests that fluorescent tags attached to the heads for stepping studies^26^,^27^ may not reliably report the position of the MT-bound stalkheads. During stepping, this flexing of the attached motor will allow the detached stalkhead of its partner motor great freedom to explore the surface of the MT to find its next binding site. This, together with extensibility between the two heads of the dimer, which will differ between the native dimer and the artificial dimers used here and elsewhere, provides a structural basis for the great range of step sizes seen in dynein stepping studies^24^,^25^,^26^,^27^,^48^. This inherent flexibility of the dynein motor, and the quasi-independent flexibility of the two motors of offset dimers, implies that it is wrong to imagine stepping dynein as having a single structure, even when both motors are attached to the MT. It will therefore be a challenge to determine the structure of any dynein-MT complex at high resolution, since current methods for this all combine data from many molecules. Dynein flexibility also raises new questions about the nature of the allosteric communication between the ATPase cycle in the head and the MT binding affinity of the stalkhead that is vital to dynein’s many cellular functions.

## Methods

### Dynein expression and purification

*D. discoideum* cytoplasmic dynein was expressed in cells derived from the *D. discoideum* Ax2 strain. The plasmid carrying the dynein gene was introduced into the *Dictyostelium* cells by electroporation, and transformed cells were selected in HL-5 medium supplemented with 10 μg ml^−1^ blasticidin S, 10 μg ml^−1^ G418 and 10 μg ml^−1^ tetracycline on culture dishes at 22 °C for 1 week. The transformed cells were grown at 22 °C with shaking until the cell density reached ∼5 × 10^6^ cells per ml. The medium was then replaced with one without tetracycline and blasticidin S to induce the expression of the recombinant dynein. After being cultured for an additional ∼24 h, the cells were collectd by centrifugation. Purification was carried out at 4 °C or on ice. The cells were lysed with sonication in PMG buffer (100 mM PIPES-KOH, 4 mM MgCl_2_, 0.1 mM EGTA, 0.9 M glycerol, 1 mM 2-mercaptoethanol, 10 μg ml^−1^ chymostatin, 10 μg ml^−1^ pepstatin A, 50 μg ml^−1^ leupeptin, 500 μM PMSF and 0.1 mM ATP (pH 7.0)) supplemented with 10 mM imidazole, centrifuged at 24,000*g* for 20 min, and the supernatant was centrifuged further at 187,000*g* for 60 min. The resulting high-speed supernatant was mixed with nickel nitrilotriacetic acid (Ni-NTA) agarose (Qiagen, Hilden, Germany) for 1 h. After the resin had been washed with PMG buffer supplemented with 20 mM imidazole, the adsorbed proteins were eluted with PMG buffer supplemented with 250 mM imidazole. Eluted fractions were supplemented with 5 mM EGTA, 0.1 mM EDTA and 150 mM NaCl and mixed with 0.5 ml of FLAG-M2 affinity gel (Sigma) for 1 h. After the resin had been washed with PMEGS buffer (100 mM PIPES-KOH, 150 mM NaCl, 4 mM MgCl_2_, 5 mM EGTA, 0.1 mM EDTA, 0.9 M glycerol, 1 mM DTT, 10 μg ml^−1^ chymostatin, 10 μg ml^−1^ pepstatin A, 50 μg ml^−1^ leupeptin, 500 μM PMSF and 0.1 mM ATP (pH 7.0)) and PMEG30 buffer (30 mM PIPES-KOH, 4 mM MgCl_2_, 5 mM EGTA, 0.1 mM EDTA, 0.9 M glycerol, 1 mM DTT, 10 μg ml^−1^ chymostatin, 10 μg ml^−1^ pepstatin, 50 μg ml^−1^ leupeptin, 500 μM PMSF and 0.1 mM ATP (pH 7.0)), the recombinant dynein was eluted with PMEG30 buffer supplemented with 200 μg ml^−1^ FLAG peptide (Sigma). The eluates were centrifuged at 100,000*g* for 15 min and the supernatant was used for further study. Purified proteins were shipped from Japan to the UK in ice or stored on ice and used within 3 days^49^,^50^.

For a monomeric wild-type motor domain construct, we used HFB380 (ref. 51). To generate dimeric GST-380 motor domains, *Schistosoma japonicum* GST coding region (nucleotides 258-917 of pGEX4T-3 vector (GenBank accession no U13855)) was inserted between the His_6_-FLAG-tag and the 380-kDa motor domain (V1388-I4730) of HF380 construct^8^ with bridging sequence GGAAAVDK between GST and dynein. As part of a larger study of stalkhead properties, we discovered stalkhead chimera constructs with enhanced MT-binding affinity (monomeric HFB380H7 and dimeric GST-380H7), by replacing the *D. discoideum* stalkhead sequence (A3372–K3495) with that of *H. sapiens* axonemal dynein heavy chain 7 (I2676–A2811; KIAA0944 in the HUGE database, Kazusa DNA Research Institute). Recombinant dynein constructs were expressed in *D. discoideum* and purified using Ni-NTA agarose (Qiagen) and anti-FLAG M2 affinity gel (Sigma-Aldrich) as described above. Negative-staining EM using 100 nM GST-380H7 (no MT) showed the preparation was all dimeric; no monomers were seen, indicating that neither GST dissociation nor proteolytic cleavage into monomers were at detectable levels.

### Single-molecule fluorescence motility assays

To observe the movement of GST-380 and GST-380H7 dimers, we genetically inserted a SNAP-tag (New England Biolabs) into the AAA2 module of the motor domain (S2476-S2477)^13^ and a biotin-tag between FLAG-tag and GST^52^. Purified SNAP-tagged GST-380 and GST-380H7 dimers (‘GST-380-SNAP’ and ‘GST-380H7-SNAP’) were fluorescently labelled with DY-647 (Dyomics) via the SNAP-tag by incubating 0.5 μM GST-380H7 with 5 μM SNAP-Surface 647 (New England Biolabs) overnight at 4 °C. The final labelling ratio of DY-647 per dynein motor domain was ∼0.5. Free DY-647 was removed by a Micro Bio-Spin chromatography column (Bio-Rad)^13^. Porcine tubulin was fluorescently labelled using Oregon Green 488 carboxylic acid succinimidyl ester (Invitrogen) following the procedure of Hyman *et al*.^53^ Pelleted MTs were resuspended at high concentration in 0.1 M Na-HEPES, pH 8.6, 1 mM MgCl_2_, 1 mM EGTA and 40% (v/v) glycerol at 37 °C, reacted for 1 h with 10 mM dye, quenched with BRB80 (80 mM K-PIPES, 1 mM MgCl_2_ and 1 mM EGTA, pH 6.8) supplemented with 100 mM K-glutamate, 40% glycerol and separated from unbound dye by centrifugation through a sucrose cushion. This was followed by two cycles of temperature dependent polymerization and depolymerisation in BRB80, collecting the pellet or supernatant respectively. MTs were prepared by incubating a mixture of 4 μM non-labelled tubulin and 40 pM Oregon Green 488-labelled tubulin with 0.2 mM Mg-guanosine-5'-((α,β) –methyleno) triphosphate (GMPCPP) at 37 °C for 30 min and then stabilized with 40 μM paclitaxel.

Single-molecule imaging was carried out at room temperature (25 °C)^50^. MTs were immobilized on a surface of the glass chamber via tubulin antibody (TU-01, Millipore). After washing the chamber with assay buffer (20 mM PIPES, 10 mM K-acetate, 4 mM MgSO_4_, 1 mM EGTA, 0.4 mg ml^−1^ casein, 10 μM paclitaxel, 1% 2-mercaptoethanol, 10 mM glucose, 85 U ml^−1^ glucose oxidase, 1,300 U ml^−1^ catalase and 1 mM ATP, pH 7.0), 100 pM of dynein dimer was introduced into the chamber in the same solution. Movement of fluorescently labelled dynein was observed under an objective-type total internal reflection fluorescence microscope (Olympus IX71 equipped with an Olympus PlanApo, NA 1.45, × 100 objective lens). The sample was illuminated by a blue laser (Showa optronics, D488C-50) or a red laser (Showa optronics, D635C-35). Images were acquired at 100 frames per second with an iXon+ back-illuminated electron multiplier charge-coupled device camera (Andor).

Movement of dynein, using the DY-647 fluorescence, was determined by a two-dimensional Gaussian fitting algorithm^54^ using the custom software^55^. Run length and duration are the total distance of movement and the total time, respectively, during a single interaction event of a dynein molecule on a MT. The mean run length (*l*_mean_), mean duration (*τ*_mean_) and mean velocity (*v*_mean_) were calculated by fitting with the cumulative distribution functions:




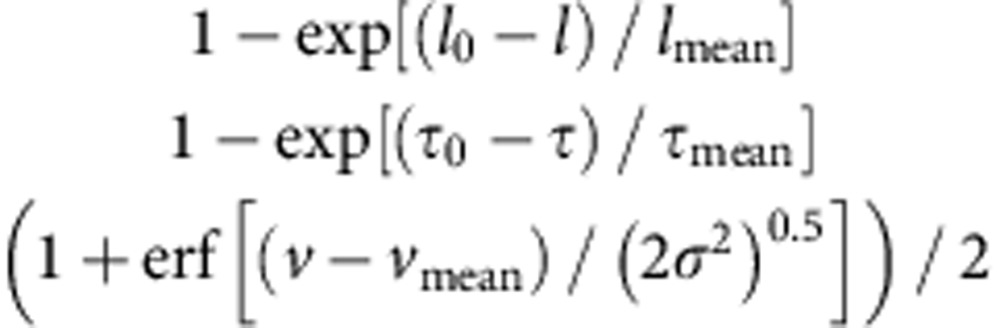




respectively, to provide binning-independent fittings^56^, where erf is the error function and *l*_0_ (lower limit for detection), *τ*_0_ (lower limit for detection) and *σ*^2^ (variance) are fitting parameters.

The duty ratio of each motor of dimeric dynein was estimated using a formalism in which the two heads are assumed to act independently^42^. Average number of steps per run was estimated from the measured average run length (600 nm; Supplementary Table 1) divided by an assumed step size. The value of duty ratio (*r*) giving the estimated number of steps was then obtained by successive approximation. We tested step sizes of 16.6, 8.3 and 4.15 nm, the last value representing an average from alternation between superposed and 8.3-nm offset heads (see Fig. 4b). The average run length corresponds to 36, 72 and 144 steps, respectively, and these would require duty ratios of 0.86, 0.90 and 0.93, respectively. Thus, duty ratio is ∼0.9, relatively insensitive to step size.

### Dynein preparation for EM

On receipt of dynein from Japan within 5 days of purification, FLAG peptide and nucleotides were immediately removed by exchanging the buffer with 10 mM PIPES-KOH (pH 7.0), 4 mM MgSO_4_ and 1 mM EGTA using a centrifugal filter device (Amicon ultra-4, 30 kDa cutoff) and the dynein was both drop-frozen and stored in liquid nitrogen. Beads were quickly thawed just before preparing EM grids.

### MT preparation for EM

Twice-cycled MAP-free porcine tubulin was from Cytoskeleton, Inc. GMPCPP was from Jena Bioscience. Tubulin in the presence of GMPCPP was polymerized using a modification of an earlier protocol^57^. Tubulin (8.1 μM) was incubated on ice in 80 mM PIPES-KOH (pH 6.8), 4 mM MgCl_2_, 1 mM EGTA and 0.64 mM GMPCPP for 20 min, then raised to 37 °C for 30 min for polymerization. To remove GMPCPP, the MT solution was centrifuged at 30,300*g* for 5 min at 35 °C, the supernatant was discarded and the pellet was resuspended in 10 mM PIPES-KOH (pH 7.0), 4 mM MgSO_4_, 1 mM EGTA and 400 μM taxol at 27 °C to give 100 μM MT (MT concentration is expressed as tubulin dimer concentration in this paper). MTs were stored in liquid nitrogen by rapid freezing and were thawed rapidly just before preparing EM grids.

### Negative stain EM of dynein in the absence of MTs

Carbon-coated copper EM grids were treated under an ultraviolet lamp for ∼10 min before applying dynein. 0.2 mM ATP was added to 15–30 nM GST-380 in 50 mM K-Acetate, 4 mM MgSO_4_, 1 mM EGTA, 10 mM PIPES-KOH (pH 7.0) on ice and immediately applied to the grid and negatively stained with 1% uranyl acetate.

To reduce changes in structure arising from prolonged adsorption time on the carbon film, we also used the ‘rapid flush’ method devised in our group (see Fig. 1 legend in ref. 58): 50–70 μl 1% uranyl acetate was drawn into the tip of a 200 μl Pipetman, the volume dial turned to draw in 5 μl air, then turned again to draw up 5 μl dynein solution, kept separate from the stain by the small air gap. The entire contents of the tip were then ejected across the face of the carbon film, allowing dynein to briefly adsorb, followed within milliseconds by fixation by uranyl acetate. Excess stain was drawn from the side of the grid by filter paper in the usual way, and the grid dried at room temperature. 0.2 mM ATP was added to dynein just before application to the grid. We used 29 nM and 58 nM GST-380 in 50 mM K-Acetate, 4 mM MgSO_4_, 10 mM PIPES-KOH (pH 7.0) or 100 nM GST-380H7 in 2.7 mM MgSO_4_, 0.67 mM EGTA and 6.7 mM PIPES-KOH (pH 7.0).

Electron micrographs were recorded with continuous illumination either using a JEOL JEM1200EX EM operated at 80 kV, nominal magnification × 40,000 on film, or using an FEI Tecnai T12 EM operated at 120 kV, nominal magnification × 30,000 using a CCD camera.

### Cryo-EM of dynein on MTs

For GST-380H7 dimeric dynein on MTs in the presence of ATP, the mixture contained 330 nM GST-380H7 dynein, 3.7 μM MT, 2.1 mM MgSO_4_, 0.53 mM EGTA, 38 μM taxol, 0.09% Tween-20, 5.3 mM PIPES-KOH (pH 7.0) and 3.6 mM ATP. The dynein was mixed with MTs without nucleotides, then ATP was added. For dense decoration of dynein on MTs (Fig. 1c), the concentration of MTs was halved.

For HFB380H7 monomeric dynein on MTs in the absence of nucleotides, the mixture contained 10–30 nM HFB380H7 dynein, 1 μM MT, 2 mM MgSO_4_, 0.5 mM EGTA, 46 μM taxol, 0.1% Tween-20 and 5 mM PIPES-KOH (pH 7.0).

Specimen (2.5 μl) was applied to a lacey carbon grid (Agar Scientific S166-4), manually blotted and frozen by plunging into liquid ethane, and subsequently stored in liquid nitrogen. The time from the addition of ATP to freezing was 36–46 s. For GST-380H7, within this time, over 3 mM total ATP would remain in the solution, based on ∼10 s^−1^ per head ATPase rate for dimeric dynein in the presence of 4 μM MT. This is calculated from steady-state MT-activated ATPase data on this dimeric dynein measured in 10 mM PIPES-KOH (pH 7.0), 50 mM K-acetate, 4 mM MgSO_4_, 1 mM EGTA, 10 μM paclitaxel and 1 mM DTT by using a coupled enzymatic assay kit (EnzChek phosphate assay kit, Molecular Probes)^13^. This yielded *k*_cat_ of 15±0.9 s^−1^ per head and *K*_m_ of 2.5±0.7 μM MT.

To prevent detachment of the motor from MTs^59^ we did not glow discharge the grids and we used manual blotting, by touching the edges of the grid then blotting a single side of the grid for up to 3.5 s using filter paper. The liquid film was typically ∼50-nm thick, since intersections between two MTs show no flattening (Fig. 1c).

The grids were transferred using a Gatan 626 holder filled with liquid nitrogen into an FEI Tecnai F20 EM with a field emission gun operated at 200 kV. The EM was operated in low-dose mode at a range of defocus (∼1.5–5.8 μm). We obtained 480 micrographs using a Gatan US4000SP CCD camera at nominal magnification × 25,000. The pixel size was calibrated as 0.454 nm using the 2.30 nm spacing of Tobacco Mosaic Virus (kind gift from Prof. Lomonossoff, John Innes Centre, UK). We used CTFFIND3^60^ to estimate the defocus of micrographs and corrected them by phase-flipping using SPIDER^61^. Image contrast was inverted (protein is pale).

### Determination of MT polarity

To determine MT polarity (Supplementary Fig. 3), we used the Tubule J software^62^. Polarity determination was made possible using tubulin polymerized in the presence of GMPCPP to strongly favour MT with 14_3 structure^63^,^64^, the polarity of which can be obtained by computational image processing^31^, independently of dynein features. GST-380H7 dynein steps robustly along such GMPCPP-polymerized MTs (Supplementary Table 1) (also ref. 57). We analysed 2,313 MTs, unambiguously determining the polarity of 1,082 14_3 MTs^31^. Briefly, (see also Supplementary Fig. 3) 14-protofilament MTs were selected by analysing the number of Moiré fringes. After computational straightening, 14_4 MTs were identified and rejected based on the spacing along the meridional direction between layer lines in the FFT at ∼4-nm spacing. MT polarity was then determined by inspecting the Moiré pattern within the Fourier-filtered image generated using only near-equatorial data (Supplementary Fig. 3c,d). Very rare 14_2 MTs were identified and rejected based on their shorter Moiré repeat distance^35^.

### Dynein particle picking

Using the EMAN 1 BOXER^65^ we manually selected 10,080 particles, which were dynein-sized lying alongside polarity-determined MTs. To select isolated dyneins on the MT (Fig. 1d,e), we used medium to high defocus (2.6–5.8 μm) micrographs, and selected from the total data set those which had only one object or two objects within 40 nm of one another, in either case with no neighbouring particles within 40 nm of these in both the plus- and minus-end directions. This produced *n*=711 isolated dyneins of which *n*=330 had only a single visible head (that is, superposed dimers) and *n*=381 had two visible heads (that is, offset dimers). To select more superposed dimers from within the remainder of the data set, we performed alignment and classification, selecting classes showing a single head not overlapped by parts of other heads. Such classes may contain single heads that are members of offset dimers with non-overlapping heads, but these are uncommon (Fig. 3), and would be expected to form their own classes due to their weaker density. This yielded a further 1,510 particles.

### Dynein-MT particle alignment

Image alignment, classification and all image analysis were performed using the SPIDER software^61^. Having determined MT polarity, we manually estimated the angle of the MT component of each particle with respect to the *x* axis in the micrographs, with MT plus end pointing left, and combined dyneins from both sides of the MT by mirroring those on one side of the MTs. Then reference-free alignment of the particles used as starting rotation angle the estimated angle of the MT within the micrograph. Initial alignment used all features for translational alignments but for rotational alignments we excluded the dynein motor itself (using an inner ring radius within the AP RA command) and included features from both edges of the adjacent MT. Reference-free alignment was performed for ten iterations. Images in which the MT was misaligned were excluded using conventional image classification techniques^30^,^66^ to identify and remove classes with tilted MTs.

Alignment of the isolated dynein particle data set (shown in Fig. 1g,h) was refined by focusing on each ring within each particle. We manually identified the centre of each ring (producing two images from each offset dimer) and masked features outside this with a soft-edged mask. This produced a total of *n*=1,092 ring images (2 × 381 offset+330 superposed) which were combined into a single image stack for subsequent reference-free translational alignment. Maintaining the rotational alignment obtained in the previous step (to ensure MTs remain horizontal) we refined their translational alignment in two successive rounds, excluding those that were assigned excessively large translations arising from spurious image features (leaving *n*=374 offset dimers and *n*=322 superposed dimers). To create class averages showing the heterogeneity of ring configurations in offset dimers (Fig. 3a) we applied classification procedures to the (*x*,*y*) coordinates of each ring.

### Pixel intensity value measurement of dynein motor domain

The dynein ring total pixel values measurement (Fig. 1f) was carried out after adjusting each image to ensure the background ice had a mean pixel value of zero. The projected sum of pixel values of each dynein dimer was then calculated as shown in Supplementary Fig. 4, measured relative to the values of the adjacent MT in each individual particle (Supplementary Fig. 4b). Images of monomeric dynein were treated identically to those of superposed dimers, except that MT polarity was not determined.

### Atomic model building

To create hypothetical atomic models of the dynein dimer strongly bound to a MT (Supplementary Fig. 6), we first fitted the tubulin dimer that is part of a murine stalk-stalkhead-tubulin dimer atomic model obtained by cryo-EM analysis (PDB 3J1T^36^) into a cryo-EM map of a 14_3 protofilament MT (emd_5194 (ref. 67)). To complete the dynein motor we used the 2.8-Å crystal structures of the ADP-dynein motor domain from *D. discoideum* lacking the stalkhead (PDB 3VKG^8^). This comprises two independent dynein monomers (chains A and B), that are arranged back-to-back in the crystal. Chains A and B also have different stalk angles relative to the ring (∼25°)^8^, which therefore repositions the rings in the MT-bound dynein models depending which chain is fused to the murine stalk. We superposed the stalk using the CCP4 LSQKAB program^68^, to fit the Cα atoms of the distal portions of CC1 and CC2 of the motor domain stalk coiled coil to the corresponding atoms of the murine stalk (Supplementary Fig. 5). To create complete molecule A, we fitted residues 3350–3366 (CC1) and 3496–3514 (CC2) in chain A of 3VKG to residues 3264–3280 (CC1) and 3409–3427 (CC2) in chain A of 3J1T (Supplementary Fig. 5b). To create complete molecule B, we fitted residues 3350–3358 (CC1) and 3502–3514 (CC2) in chain B of 3VKG to residues 3264–3272 (CC1) and 3415–3427 (CC2) in chain A of 3J1T (Supplementary Fig. 5c). We then measured the distances between Cα atoms among the fitted residues. At the shortest distance among the fitted residues we fused the two structures together.

This produced two alternative back-to-back models, in which necessarily only one of the stalks was attached to the MT (Supplementary Fig. 6b,c). In both these models the stalks are not superposed in the view seen in our data. Therefore we made a third back-to-back model by rotating chains A and B about an axis approximately parallel to the stalks until the stalks superposed (Supplementary Fig. 6a).

To make four front-to-back models of dimeric dynein on the MT (Supplementary Fig. 6d–g), we deleted the detached dynein motor in the back-to-back models shown in Supplementary Fig. 6b,c. We then docked a second complete dynein molecule, either A or B, to a neighbouring tubulin dimer in an adjacent protofilament, by fitting the model into the MT cryo-EM map as above. To rotate each ring into the plane of view (see text), we broke the coiled-coil chains near the stalk-stalkhead junction.

Molecular graphics were created using the UCSF Chimera package^69^.

### Deduction of dynein mechanical properties

The torsion spring constant for the dynein stalk-stalkhead hinge was estimated by applying the Equipartition Theorem to the observed distribution of stalk angles, using *κ*=*k*_B_*T*/*σ*^2^, where *κ* is the torsion spring constant (N m rad^−2^), *k*_B_ the Boltzmann constant, *T* absolute temperature (293 K in our case) and *σ*^2^ the variance of measured lever angle (rad^2^). To express stiffness as the apparent cantilever stiffness (*k* in pN nm^−1^) of the MT-attached stalk (that is, the resistance experienced by a force applied parallel to the MT axis at the base of the stalk, where the linker ends), we equated the energy for rotation of the stalk through an angle *θ* (*κ*.*θ*^2^/2) with the energy to bend the stalk to move the head through the same distance (*x*) as produced by the angular rotation (that is, *kx*^2^/2). For a cantilever, length *L*, at 90° to the MT axis, *x*=*L*.sin*θ*. Hence, for small displacements for which sin*θ*≈*θ*, *k*=*κ*/*L*^2^. When the cantilever is at angle *φ* (in our case *φ* ≈42°), the effective lever length is *L*.sin*φ*, hence *k*=*κ*/(*L*.sin*φ*)^2^. We estimated *L*=12.3 nm by subtracting the head radius (6.5 nm (ref. 8)) from our measured mean value (18.8 nm) of the distance from ring centre to hinge.

To observe trends in mean stalk angle due to increasing intramolecular tension in offset dimers with increasing stalkhead separation (Fig. 3e,f), we generated running averages as follows. Using data sorted by stalkhead separation, the mean angle and mean stalkhead separation of the first 100 data points was calculated. The first data point was then removed, the 101st data point was added and the calculations repeated. The cycle was repeated until all data points had been included.

## Additional information

**How to cite this article:** Imai, H. *et al*. Direct observation shows superposition and large scale flexibility within cytoplasmic dynein motors moving along microtubules. *Nat. Commun.* 6:8179 doi: 10.1038/ncomms9179 (2015).

## Supplementary Material

Supplementary Information Supplementary Figures 1-9, Supplementary Table 1 and Supplementary References

Supplementary Movie 1 Comparing GST-380 and GST-380H7 binding to MTs in ATP. Total Internal Reflection Fluorescence Microscopy (TIRF-M) shows pink spots of DY-647-labelled GST-380-SNAP (left) or DY-647-labelled GST-380H7-SNAP (right) moving on GMPCPP-MTs (green) in the presence of 1 mM ATP and 0.5 nM dynein dimers. Frames were recorded at 20 frame s-1 (i.e. 50 ms per frame) and presented here 5 times faster than actual speed. The width and height of the fields shown is 71.68 μm (512 pixels). Few GST-380 molecules bind, whereas dense binding of GST-380H7 almost obscures the MT fluorescence.

Supplementary Movie 2 Single molecule motility of GST-380H7 dimers along GMPCPP-MTs. Total Internal Reflection Fluorescence Microscopy (TIRF-M) shows DY-647-labelled GST-380H7-SNAP (pink spots) moving processively on GMPCPP-MTs (cyan) in the presence of 1 mM ATP. Frames were recorded at 6.7 frame s-1 (i.e. 150 ms per frame) and presented here at 30 frame s-1 (i.e. ~4.5 times faster than actual speed). The width and height of the field is 27.2 μm (170 pixels).

Supplementary Movie 3 Dynein flexibility in superposed dimers on MT. Ten frames of GST-380H7 class averages, using data from the full data set, show that the major site of stalk flexibility is at the stalk-stalkhead junction. The field is 33 nm wide and 36 nm high.

Supplementary Movie 4 Atomic model of superposed dimer on MT. Model shown in Fig. 2h coloured according the same scheme, with the invariant prolines at the stalkstalkhead junction depicted in red.

Supplementary Movie 5 Dynein flexibility in offset and superposed dimers on MT. 13 frames of GST-380H7 class averages are shown, using data from the stringently defined dimers. The heads move along a wide arc centred on the stalk-stalkhead junction, so the movement exhibits both axial and radial components with respect to the MT. Left panel shows offset dimer trailing heads; middle panel shows offset dimer leading heads; right panel shows superposed heads. Note the similarity in flexibility, including that the ring is seen face-on throughout. Also note the greater visibility of the stalk in the superposed dimer, consistent with it comprising two superposed stalks. Each movie field is 91 nm wide and 77 nm high.
