# Supplementary material for: Direct observation shows superposition and large scale flexibility within cytoplasmic dynein motors moving along microtubules
Source: Nat Commun. 2015 Sep 14;6:8179. doi: 10.1038/ncomms9179 (PMC4579568; doi:10.1038/ncomms9179)
Supplement: Supplementary Information — Supplementary Figures 1-9, Supplementary Table 1 and Supplementary References [file ncomms9179-s1.pdf]

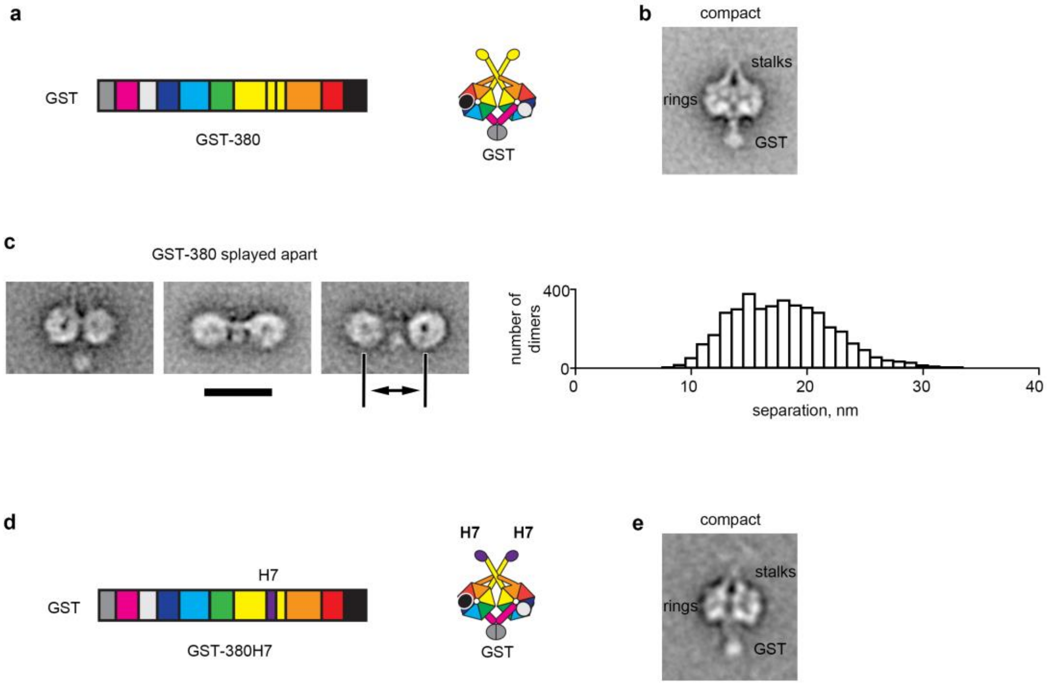

Supplementary Figure 1. Electron microscopy of dimeric dynein in the absence of MT. Recombinantly expressed motor domains dimerised by N-terminally fused GST imaged in the presence of 200 μM ATP but without MT, using negative stain electron microscopy and single-particle image processing. Two different constructs are shown, GST-380 (**a**-**c**) and GST-380H7 (**d**, **e**). (**a**) GST-380 primary sequence diagram and cartoon depicting the resulting dimer structure. Colour coding is identical to Fig. 1a. (**b, c**) GST-380 in 200 μM ATP showing both compact (**b**) and splayed apart (**c**) structures. In the compact structure (**b**) the GST dimer protrudes opposite the stalks indicating that the linkers are in a conformation similar to the primed conformation. (**c**) Three examples of classes showing differently splayed-apart dimers and measurement of the separation between the two rings (arrow). The resulting histogram (right) shows a maximum head-head separation of 40 nm with 99% less than 30 nm apart. The mean separation is 17.3 nm (± 4.3 nm S.D., *n*=4,030). (**d**) GST-380H7 primary sequence diagram and cartoon of the dimer with replaced stalkhead (3372-3495) from human dynein 7 (DNAH7), as used in the MT stepping study. (**e**) The compact structure of GST-380H7 in 200 μM ATP. The compact structures (**b**, **e**) appear two-fold symmetric about an axis running up the page with paired stalks suggesting the heads adopt either a back-to-back or a front-to-front configuration, unlike the situation in the superposed MT-bound dimer (which is front-to-back). This structure is reminiscent of so-called 'phi-particles' reported previously in tissue-purified porcine dynein[1] and may be an inactive conformation of dynein[2]. Scale bar in (**c**) for (**b**, **c** & **e**) is 20 nm.

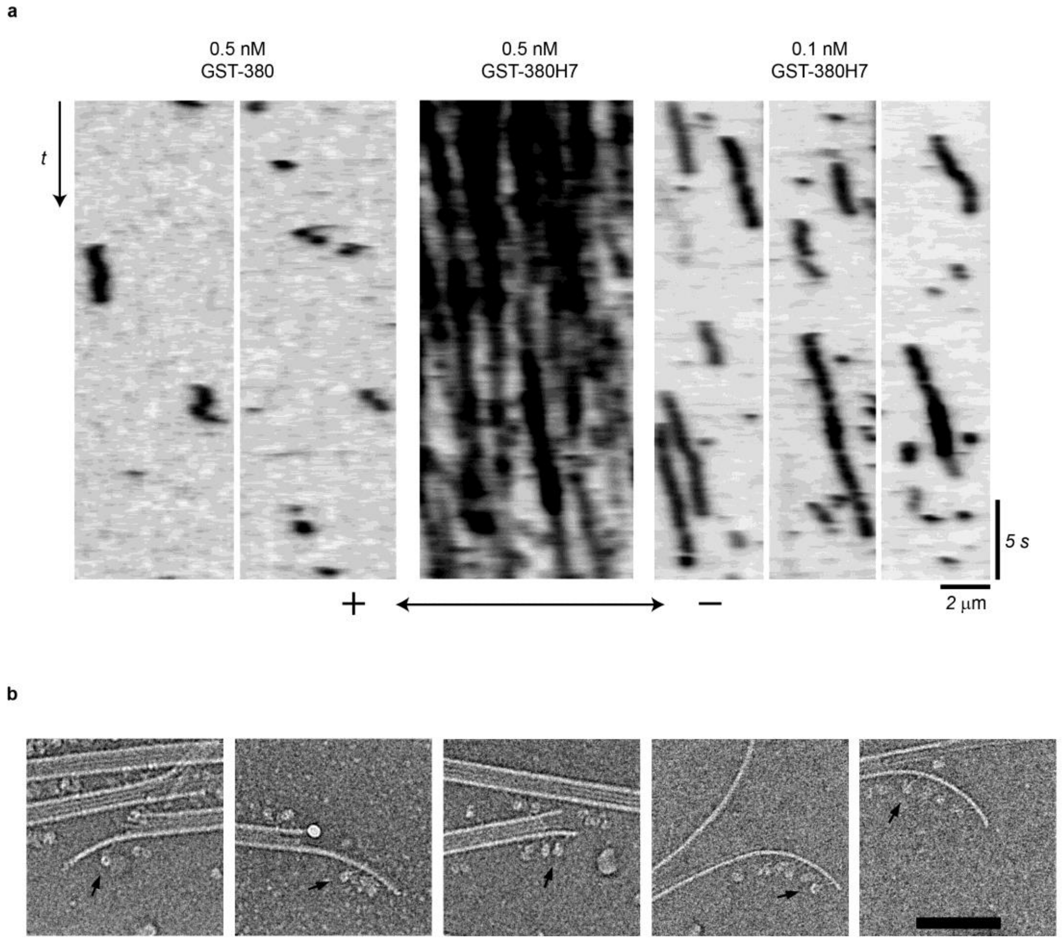

Supplementary Figure 2. Behaviour of GST-dyneins on MTs in the presence of Mg-ATP.
(**a**) Kymographs of fluorescently-tagged GST-380 and GST-380H7 moving on MT in single molecule motility experiments (see Supplementary Table 1 and Methods). Contrast is inverted so that black lines in the kymographs correspond to the dynein molecules. Minus end of MT is to the right. (**b**) Gallery of examples of flared MT ends showing GST-380H7 dynein dimers attached in the presence of ~2 mM Mg-ATP (arrows) and imaged by cryo-EM. The strongly angled stalks indicate the polarity of the MT in each case (minus end to the right). The leftmost panel thus appears to show flaring at the plus end of the MT and the other examples show flaring at the minus end. Dynein dimers attached to flared MT ends were not analysed in this study. Scale bar 100 nm.

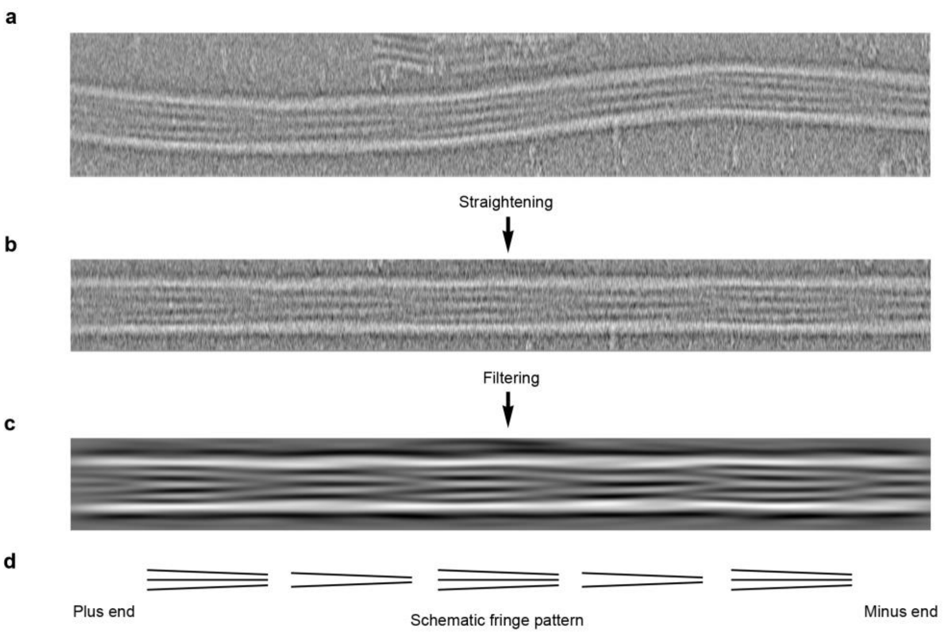

Supplementary Figure 3. Method of MT polarity determination using the Tubule J software[3].
For clarity, images are shown compressed parallel to the MT axis 4-fold. Width of window is 1.63 µm. (**a**) A 14-protofilament 3-start helix (14_3) MT is shown. The MT image was cut out from a raw cryo-electron micrograph and (**b**) computationally straightened. (**c**) The Fourier filtered image shows a Moiré pattern, drawn below in (**d**) for clarity. The 3-0-2-0 repeating sequence in the Moiré pattern indicates this is a 14-protofilament MT. The tapering feature of the Moiré pattern pointing to the right, confirms the polarity: the narrower end is towards the minus end of the MT[4].

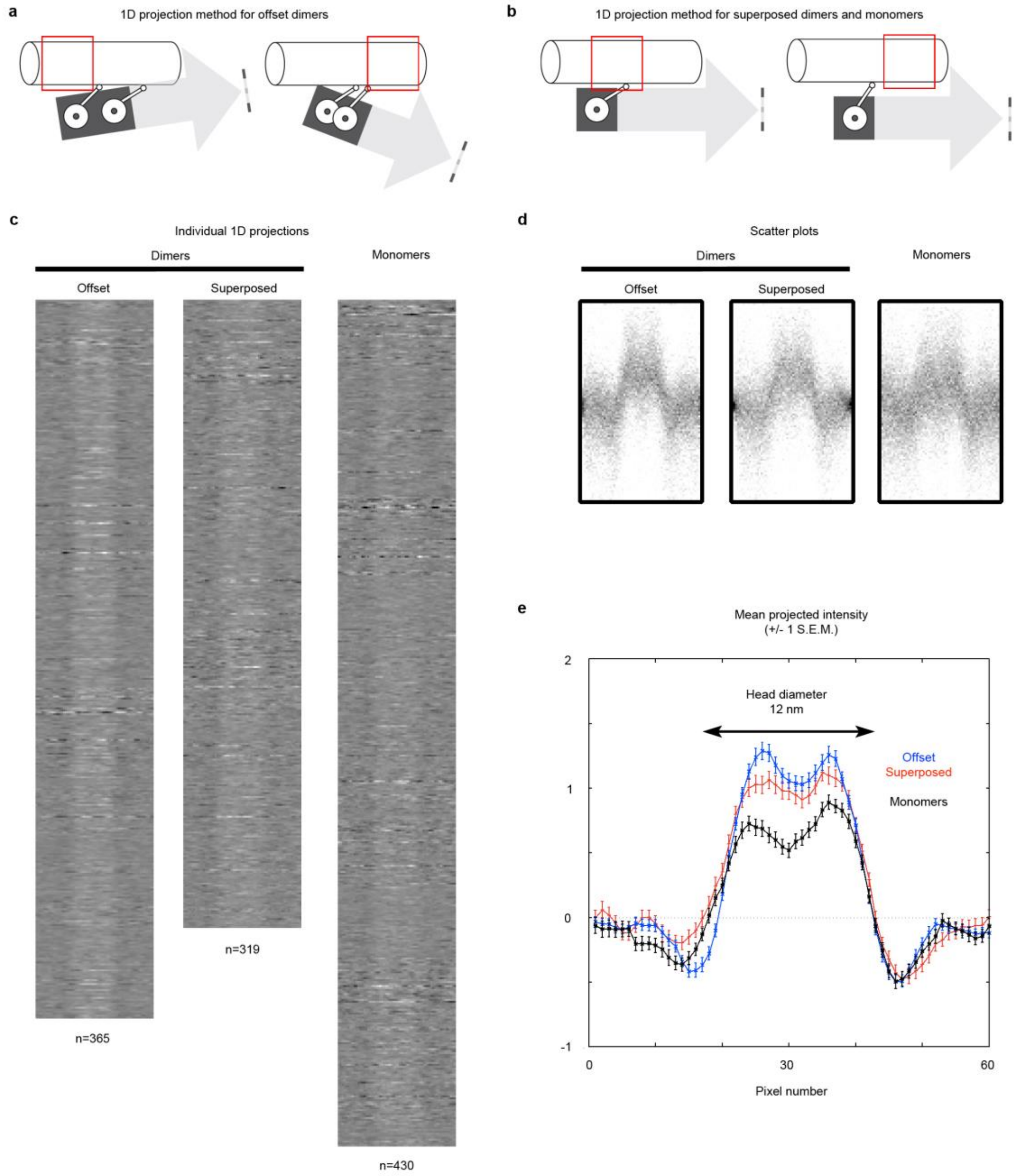

Supplementary Figure 4. Pixel value measurements of offset and superposed dimers compared to monomeric dynein.

(**a, b**) Cartoons depicting the one-dimensional (1D) projection method used to determine the head pixel values of offset (**a**) and superposed (**b**) dimers relative to the MT track. Monomers are measured as in (**b**). Two examples are shown in each case. (**a**) For offset dimers the projection direction (grey arrow) passes through the centres of both rings regardless of their separation or disposition relative to the MT track and (**b**) for superposed dimers and monomers through the centre of the ring and parallel to the MT axis regardless of its distance from the MT track. The 2D projected area is shown by the dark grey box. The MT region used to normalize each measurement is illustrated by the red box. Its position is chosen in each case from a selection of three alternative locations along the MT, using the one which has the middle summed pixel value of the three to avoid a spurious value arising from ice contamination or other irregular features. (**c**) Individual 1D projections stacked in columns show a central bright vertical stripe corresponding to the heads. (**d**) Scatter plots combining all individual 1D projections. (**e**) The mean pixel-by-pixel pixel value measurements of the 1D projections (± 1 sem) show a broad peak corresponding to the diameter of the head (shown by the arrow) with a central drop in value corresponding to the central channel. The head is fringed by negative peaks remaining after phase flipping the contrast transfer functions of the micrographs. Superposed dimers have similar values to offset dimers and twice the value of monomers. The results are summarized in Fig. 1f.

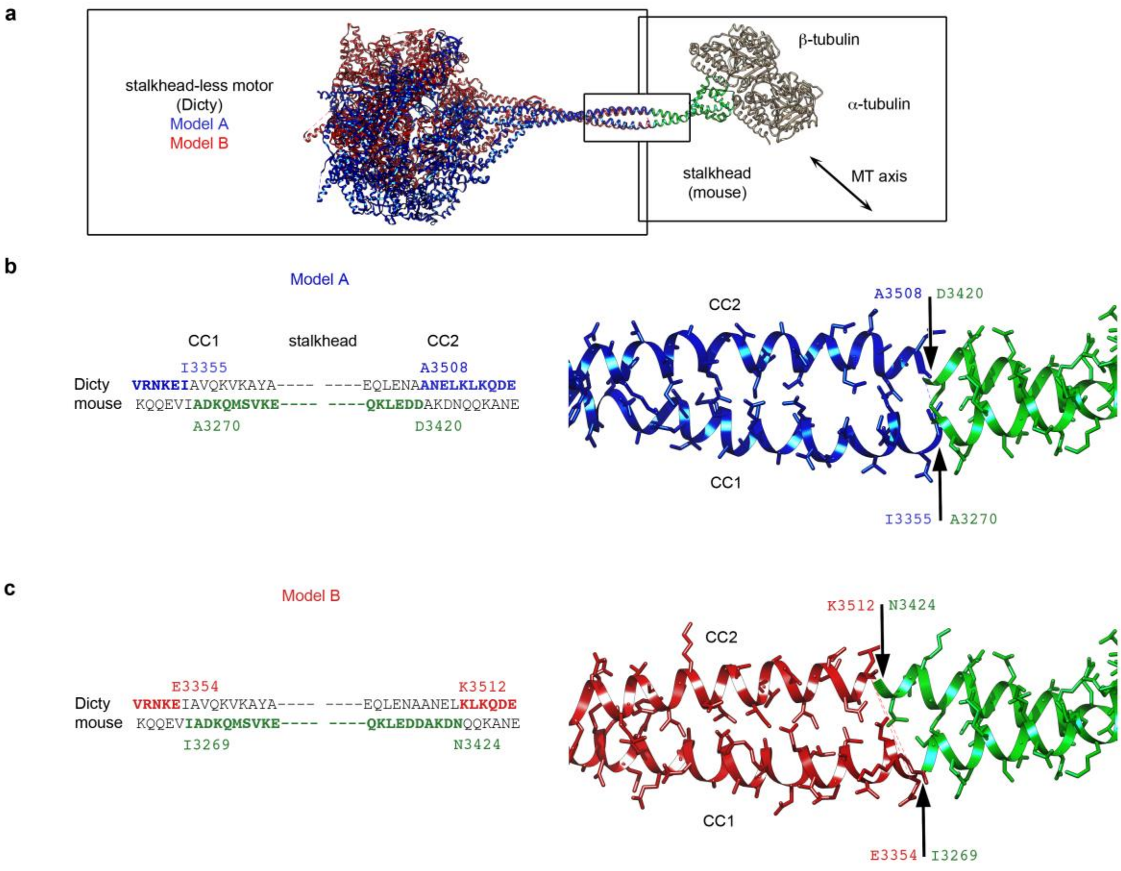

Supplementary Figure 5. Creation of initial models of monomeric dynein.
(**a**) Two models of the stalkhead-less *D.d.* dynein motor domain (PDB accession code 3VKG, blue[5]: chain A, red: chain B) are fused to a model of the tightly bound mouse stalkhead structure (PDB: 3J1T, green)[6]. MT axis lies in the plane of the page. This produces a stalk angle of ~45° to the MT surface. Fusion is made within the overlapping coiled coil structures (central boxed region), which share the same (α) registry, after aligning the amino acid sequences. (**b**, **c**) Details of the fusion sites in the sequences and structures are shown. The residues included in the models are shown in bold in each. Slight differences in the stalk structures between Models A and B require that the fusion sites are different in the two models. See Methods for further details. These models, docked onto a MT structure, are shown in Fig. 2g-j and Supplementary Figs 6 and 7.

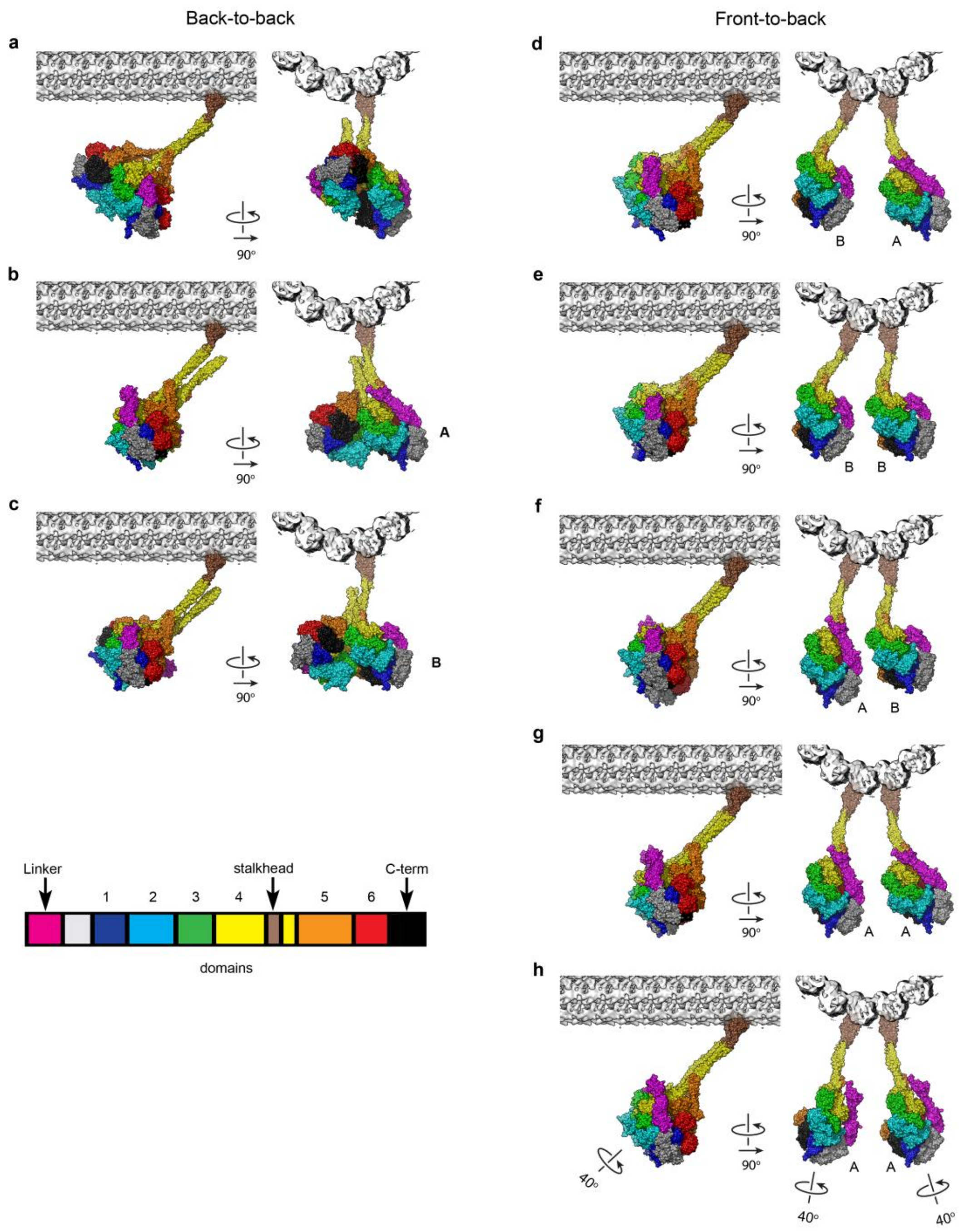

Supplementary Figure 6. Comparison of dynein models bound in pairs onto MT.
Dynein models (see Supplementary Fig. 5) bound to the MT in a variety of configurations. Side view (left) and end-on view looking towards the minus end of the MT (right) are shown in each case. (**a-c**) Back-to-back arrangement of the two models (A and B) in three different orientations bound to the MT by one of the stalkheads (the other stalkhead is pointing in the wrong direction to bind the MT in this way). In (**a**) the stalks superpose, in (**b, c**) the motors superpose with (**b**) model A nearer the observer and (**c**) model B nearer the observer. (**d-h**) Front-to-back arrangement of the two models (A and B) bound to nearest tubulin dimers on adjacent MT protofilaments in different combinations as follows: (**d**) B and A, (**e**) B and B, (**f**) A and B, (**g**) A and A. In each case the rings are found to be tilted with respect to a plane passing through the MT long axis and the stalk. (**h**) Modified dimer model in which two A models are rotated by 40° about an axis passing through the long axis of the stalk, shown by the rotation symbols. This orients the rings parallel to a plane passing through the MT long axis and the stalk. Colour key for the motor domains shows the six AAA+ domains (numbered 1-6), the stalkhead and adjacent stalk up to the site of fusion (see Methods; brown), the proposed moving part of the linker (magenta), the fixed part (grey) and the C-terminal domain (black).

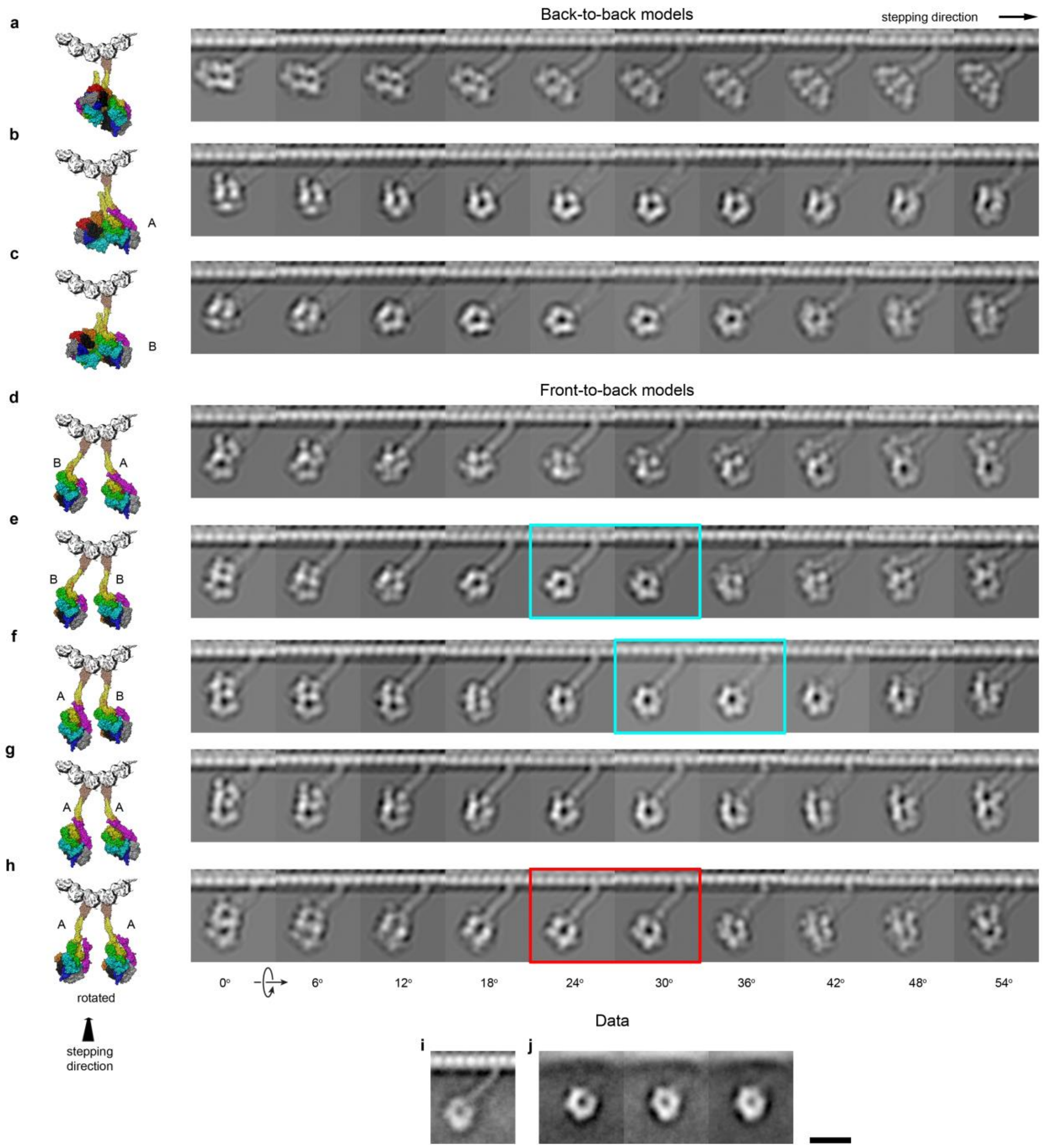

Supplementary Figure 7. Comparison of models to EM data.
Comparison between alternative superposed dimer models (**a-h**) and our data (**i**, **j**). (**a-h**) Eight different 3D models of dimers (shown in Supplementary Fig. 6, and indicated in the cartoons in end-on views on the left) are shown projected into 2D at a range of angles (at 6° intervals) around the MT long axis and with a simulated Contrast Transfer Function applied with a defocus of 4 µm. Model projections of three differently docked back-to-back models (**a-c**) and five front-to-back models (**d-h**) are shown. A similar overall appearance of the stalks and heads to our data are seen in front-to-back configurations (cyan boxes, **e, f**) but not to back-to-back configurations because head superposition and stalk superposition require different rotations around the MT axis. (**h**) The projected head shape in the model in which the heads are rotated around the stalk coiled coil axis also shows a close similarity to our data (red box). (**i**) Example class of superposed dimer and (**j**) three head classes for comparison to these models and the model heads, respectively. Scale bar (**a-j**) 16 nm.

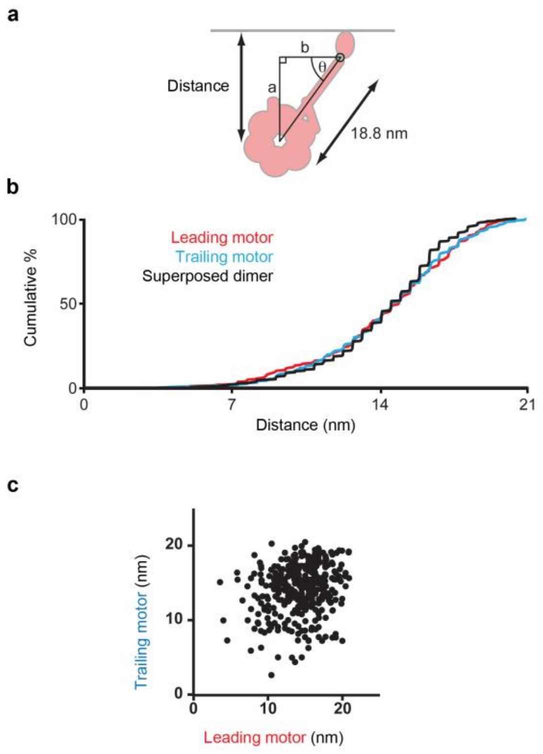

Supplementary Figure 8. Variable separation of dynein ring from MT surface.
(**a**) Cartoon depicting the measurement of distance between ring centre and MT surface, which is plotted in (**b**). Also shown are parameters used in trigonometric deduction of stalk angle and stalkhead position. This assumes that the dynein head and stalk move in the plane of the image as a rigid body about the stalk-stalkhead hinge. The distance between ring centre and hinge, measured in class averages of superposed dimers is 18.8 ± 0.45 nm; SD, *n*=11. The hinge is 2.0 nm from the MT surface, measured as the fulcrum position of flexion seen in Supplementary Movie 3. (**b**) Cumulative plots of ring-MT distance for leading (red; mean 14.3 nm ± 3.4 nm SD), and trailing (cyan; 14.4 nm ± 3.2 nm) offset motors (n=359), and superposed dimer (black; 14.2 nm ± 2.8 nm (*n*=318)). (**c**) Scatter plot of ring-MT distance of the two rings of offset dimers, showing the great independence of the two motors.

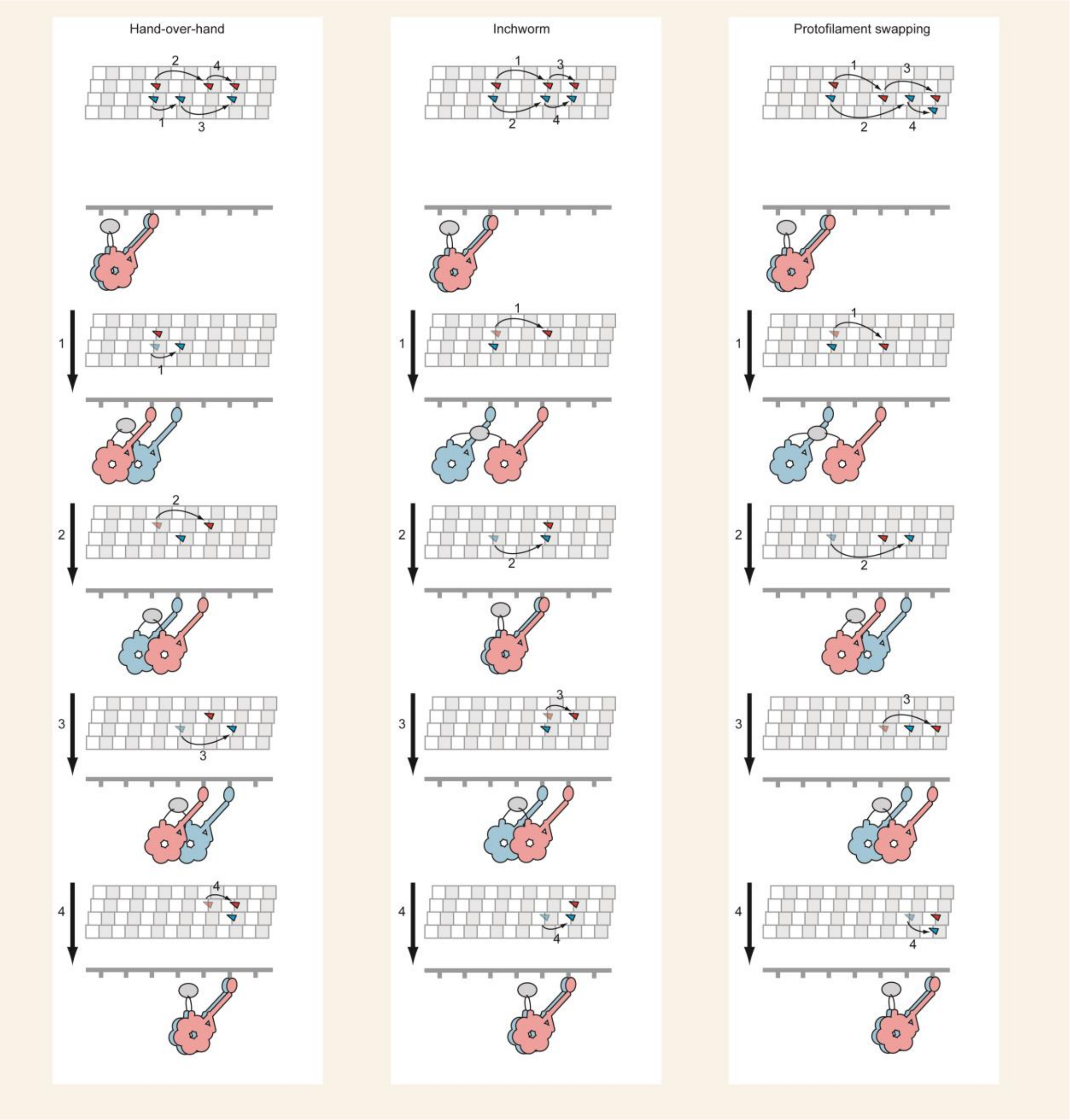

Supplementary Figure 9. Examples of stepping patterns of dimers along MT.
Example stepping patterns showing three different features of stepping: hand-over-hand; inchworm; and protofilament swapping, including stepping along a single protofilament. Stepping is to the right. At the top of each column, the diagram of 4 MT protofilaments shows the sequence of sites occupied by stalkheads on the MT surface; similar diagrams further down indicate the stalkhead movement occurring at each numbered step. The dimer cartoons show corresponding views, as would be seen in our data, at each stage in the sequence that both stalkheads are attached. The MT surface and its dynein binding sites are shown diagrammatically as a horizontal line with tick marks at 8.3 nm intervals. These cartoons are shown with the near head (pink) remaining closer to the observer, though swapping may be possible.

Supplementary Table 1. Single-molecule motility of dimeric dyneins on GMPCPP-MT in ATP.

Parameter	GST-380-SNAP dynein	GST-380H7-SNAP dynein
Velocity<br>(nm s^{-1})	610 ± 20 (*n*=78)	190 ± 20 (*n*=151)
Run length<br>(nm)	790 ± 70 (*n*=56)	600 ± 70 (*n*=96)
Duration<br>(s)	1.0 ± 0.6 (*n*=91)	5.4 ± 0.6 (*n* = 240)
Attachment rate<br>(molecule s^{-1} μm^{-1} nM^{-1})	0.05 ± 0.03 (*n*=11)	0.16 ± 0.02 (*n*=24)

Values are mean ± sem.

Supplementary References

1 Amos, L. A. Brain dynein crossbridges microtubules into bundles. *J. Cell Sci.* **93,** 19-28 (1989).

2 Torisawa, T. *et al.* Autoinhibition and cooperative activation mechanisms of cytoplasmic dynein. *Nat. Cell Biol.* **16,** 1118-1124 (2014).

3 Blestel, S., Kervrann, C. & Chrétien, D. A Fourier-based method for detecting curved microtubule centers: application to straightening of cryo-electron microscope images. *Proc. IEEE Int. Symp. Biomed. Imaging* 298-301 (2009).

4 Chrétien, D., Kenney, J. M., Fuller, S. D. & Wade, R. H. Determination of microtubule polarity by cryo-electron microscopy. *Structure* **4,** 1031-1040 (1996).

5 Kon, T. *et al.* The 2.8 angstrom crystal structure of the dynein motor domain. *Nature* **484,** 345-350 (2012).

6 Redwine, W. B. *et al.* Structural basis for microtubule binding and release by dynein. *Science* **337,** 1532-1536 (2012).
